# Intentional self harming in adolescents case report

**DOI:** 10.1192/j.eurpsy.2026.11918

**Published:** 2026-07-31

**Authors:** Z. Barac, S. Kalac, T. Brandmajer

**Affiliations:** 1 psychiatric office “Duša”; 2 psychiatry, KCCG, Podgorica, Montenegro

## Abstract

**Case report:**

Our patient is 16.2 years old, she is in the second grade of a high school that was not her choice but her mother’s choice.

She has two older sisters who are 14 and 12 years older, and the four of them live in a joint household.

The father died when the patient was four years old.

From the beginning of his schooling, she achieves very good success.

Ever since she became aware of herself, she feels dissatisfied, incomplete with occasional impressions of worthlessness and aimlessness.

From the age of 13, she started to self-harm with a nail bit, mostly on her thighs and left upper arm.

Until six months ago, no one from the house noticed, only when she cut herself deeper with a bruise and got scared and told her mother.

Since then, she has been included in psychotherapy treatment.

Self-injury is a term that encompasses all self-injury, regardless of intent.

Non-suicidal intentional self-injury differs from other behaviors due to etiological, functional, and predictive factors that are unique to this type of self-injury.

In the paper, the authors discuss the aforementioned behavior and its characteristics vs suicidal pining

**Disclosure of Interest:**

None Declared

